# Psychometric evaluation of the Chinese version of the fear of pregnancy scale: a translation and validation study

**DOI:** 10.3389/fpubh.2024.1364579

**Published:** 2024-02-22

**Authors:** Chunyan Wu, Jian Zhang, Lei Zhao, Yanhong Li, Yuanyuan Yan, Yue Wei, Zhixia Zhang, Shuming Guo

**Affiliations:** ^1^Department of Nursing, Shanxi Medical University, Taiyuan, China; ^2^Linfen Central Hospital, Linfen, China; ^3^Department of Nursing, Jinzhou Medical University, Jinzhou, China

**Keywords:** fear of pregnancy, women of childbearing age, cross-cultural adaptation, factor analysis, psychometric evaluation

## Abstract

**Introduction:**

Many women experience fear toward pregnancy, which can impact their desire to have children and the national birth rate. Thus, assessing women’s fear of pregnancy is of great importance. However, there is currently no specialized tool for assessing women’s fear of pregnancy in China. The purpose of this study is to translate the Fear of Pregnancy Scale into Chinese and test its reliability and validity among women of childbearing age.

**Methods:**

Using convenience sampling combined with a snowballing method, a cross-sectional survey was conducted on 886 women of childbearing age in two cities in China. The translation was strictly carried out according to the Brislin model. Item analysis, validity analysis, and reliability analysis were employed for psychometric assessment.

**Results:**

The Chinese version of the Fear of Pregnancy Scale comprises 28 items. Exploratory factor analysis extracted four factors with a cumulative variance contribution rate of 72.578%. Confirmatory factor analysis showed: NFI = 0.956, CFI = 0.986, GFI = 0.927, IFI = 0.986, TLI = 0.985, RMSEA = 0.032, and χ2/df = 1.444. The scale’s Cronbach’s α coefficient is 0.957, split-half reliability is 0.840, and test–retest reliability is 0.932.

**Conclusion:**

The Chinese version of the Fear of Pregnancy Scale possesses robust psychometric properties and can assess the degree of pregnancy fear among Chinese women of childbearing age. It provides a reference for formulating relevant policies in the prenatal care service system and implementing targeted intervention measures.

## 1 Introduction

Pregnancy is a special experience that most women will have to go through sooner or later. As a subjective experience, it involves a plethora of psychological changes in addition to physiological ones. For most individuals, this period is marked by expectations (1), yet it is not devoid of inevitable panic and worry (2). The anticipation is centered around the arrival of a new life and the embarkation on a novel stage of life. However, the inherent risks and uncontrollability associated with childbirth (3), coupled with potential alterations in body image (4), the transformation of roles in work and family settings (5), and economic pressures (6), may create hesitancy in women considering pregnancy.

Tokophobia, a profound fear and anxiety toward pregnancy and childbirth, was first mentioned in a qualitative study conducted in the United Kingdom involving 26 women. This condition is categorized into primary and secondary tokophobia. Women suffering from this phobia may opt not to have children (7). However, fear of pregnancy can be seen as merely one aspect of the complex psychological state women may face during the reproductive process, encompassing apprehensions and fears about various physical, psychological, and social challenges that may arise during pregnancy. Yasemin et al. define fear of pregnancy as a condition where a woman believes that her health and life could worsen due to pregnancy, feels unprepared for pregnancy, and experiences anxiety and fear about becoming pregnant (8).

As a psychological disorder, fear of pregnancy often has complex etiologies. The uncertainty surrounding the pregnancy process (3), traumatic memories from childhood, fear of childbirth pain, lack of knowledge (9), and previous negative pregnancy experiences (10, 11) can all contribute to the development of tokophobia. Consequently, in dealing with pregnancy, some choose to overcome their fears, while others opt to evade childbearing altogether. The fear of pregnancy might lead women to avoid becoming pregnant, delay pregnancy, or terminate pregnancy, thereby never taking the risk of experiencing it (12, 13). In the long term, this can reduce the birth rate across society, impacting societal development (14). This fear is not only prevalent globally but also exhibits diversity across different cultures, ages, and socioeconomic backgrounds. Particularly in China, with societal advancement and the elevation of women’s status, balancing pregnancy with career development (15), personal health (16), and social responsibilities has become a significant issue. These factors, in concert, may lead to specific manifestations of fear of pregnancy among Chinese women of childbearing age.

The ‘Outline for Women’s Development in China (2021–2030)’ notes that due to women’s unique physiological characteristics, and the responsibilities and obligations they shoulder in family and society, women are prone to psychological abnormalities. This is especially true during critical stages of a woman’s life, such as adolescence, pregnancy, and menopause. Therefore, early identification and assessment of women’s fear of pregnancy is crucial in reducing negative emotions like anxiety and depression, enhancing their overall well-being and quality of life. It also forms the foundation for pregnancy healthcare service institutions to formulate relevant policies and regulations, and to implement effective intervention measures.

Most previous studies have focused on childbirth fear and anxiety during pregnancy among pregnant women (10, 17–19), with only a few investigating the desire for pregnancy in non-pregnant women, including in China (20–23). However, the desire to give birth should not be equated with the fear of pregnancy. Studies have shown that women who have never been pregnant can experience fear of pregnancy (7, 12, 24, 25). Research on assessing this fear is limited. The ‘Childbirth Fear - Prior to Pregnancy Scale’ (CFPP), developed by Canadian scholar Stoll, is used to measure childbirth fear in young adults planning for children. It includes fears related to pain and uncontrollability, complications, and postpartum bodily changes (26). Although its target demographic is young men and women, we believe its focus is still on the state during and after childbirth, not addressing the perspective of women not currently pregnant toward the state of pregnancy itself. Recently, Turkish researchers Yasemin and Kevser developed the ‘Fear of Pregnancy Scale’ (FOPS), based on relevant literature and qualitative interviews with women of childbearing age. This scale assesses fear of pregnancy in aspects of physical appearance, maternal–infant health, spouse relationships, and daily activities. It is the first instrument specifically designed to evaluate the fear of pregnancy among women of childbearing age. It has been proven to have good reliability and validity in the Turkish population (8), but studies on its reliability and validity in other countries, especially in China, have not yet been reported.

The objective of this study is to introduce the Turkish version of the Fear of Pregnancy Scale (FOPS) to China, adapt it culturally to Chinese, and test its reliability and validity. This will create a scientific tool that is sensitive to Chinese cultural nuances. The aim is to provide a foundation for a better understanding and assessment of the current state of pregnancy fear among Chinese women and to support future interventional research.

## 2 Methods

### 2.1 Design and participants

This study was conducted from October to December 2023, aiming to assess the extent of pregnancy fear among Chinese women of childbearing age and to measure their psychometric characteristics through a cross-sectional study. The sample size was determined based on the general principles of factor analysis procedures, which require at least 10 participants per item, allowing for a larger sample size (27). For this study, with 28 items on the Chinese version of the Fear of Pregnancy Scale and anticipating a 10% rate of invalid questionnaires, the minimum sample size needed was 308 individuals. Inclusion criteria were: (a) women of childbearing age between 15 and 49 years; (b) capable of normal communication and having a certain level of reading ability; (c) agreement to participate in the study. Exclusion criteria were: (a) individuals with severe mental illnesses; (b) those already participating in similar studies. This study employed a combination of convenience sampling and snowball sampling methods to recruit participants from communities in Linfen City, Shanxi Province, and Jinzhou City, Liaoning Province. Initially, researchers utilized convenience sampling to select specific communities and contacted community leaders for both online and offline recruitment. Face-to-face surveys were conducted in community offices. Subsequently, participants recruited through convenience sampling were tasked with forwarding pre-designed electronic questionnaires to their eligible friends via their social networks, thereby implementing the snowball sampling approach. In total, 886 participants were recruited for the study.

### 2.2 Instruments

#### 2.2.1 General information

General demographic information was self-determined based on literature review and team discussions, including age, household registration, educational level, occupation, marital status, only child or not, medical insurance and average monthly earnings.

#### 2.2.2 Fear of Pregnancy Scale

The Fear of Pregnancy Scale (FOPS) was developed by international scholars Yasemin and Kevser based on relevant literature and qualitative interviews with women of childbearing age. It includes four factors: physical appearance, maternal–infant health, spouse relationships, and daily activities, with a total of 30 items. This scale is designed to assess the fear of pregnancy in women of childbearing age. It employs a self-assessment method using a Likert 6, ranging from 0 to 5, representing ‘not afraid at all’ to ‘extremely afraid.’ The total score ranges from 0 to 150, with higher scores indicating a more severe fear of pregnancy. After development, the scale was tested in a group of 398 women aged 18–45, showing a Cronbach’s alpha coefficient of 0.951 for the overall scale, and 0.868–0.935 for the individual factors (8). During cross-cultural adaptation, items were merged, and item analysis led to some reductions, resulting in a final Chinese version of the FOPS with four factors and 28 items.

### 2.3 Procedure

#### 2.3.1 Scale translation procedure

After obtaining authorization from Professor Yasemin, we rigorously followed the Brislin model (28) for the translation and back-translation of the scale. The first step involved translation: two bilingual individuals with Chinese as their native language translated the original scale from English to Chinese, and one individual fluent in Turkish and Chinese translated the Turkish version into Chinese. After discussion and modification by the research team, a consensus was reached to form the initial Chinese version of the FOPS. The second step was back-translation: two Chinese students studying abroad and proficient in English back-translated the Chinese version into English, and a native Turkish individual fluent in both Chinese and English back-translated the Chinese version into Turkish and English. Following team discussion and modification, the English and Turkish back-translated versions of FOPS were formed. The third step involved original author review: the Chinese translation, along with the English and Turkish back-translated versions, were sent to Professor Yasemin via email for review. Based on Professor Yasemin’s feedback, the back-translated and translated drafts were modified, resulting in the initial draft I of the Chinese version of FOPS. The fourth step was expert adaptation: we invited 12 experts from obstetrics, psychology, and nursing fields for cross-cultural adaptation of the initial Chinese draft I, making it more consistent with Chinese linguistic expressions, resulting in the initial draft II. The fifth step was a pilot study: 20 participants meeting the inclusion and exclusion criteria were selected for a pre-survey to assess the clarity and cultural appropriateness of the Chinese version of the scale from the perspective of the participants, thereby completing the translation process to form the Chinese FOPS.

#### 2.3.2 Data collection procedure

This study employed a combination of convenience sampling and snowball sampling methods for conducting surveys in Linfen City, Shanxi Province, and Jinzhou City, Liaoning Province. Initially, convenience sampling was used, where trained researchers engaged in face-to-face interactions with participants at community offices. Subsequently, snowball sampling was applied, expanding the reach to more women of childbearing age through the social networks of these initial participants.

### 2.4 Data analysis

Data entry was double-checked by two individuals using Excel 2021. Descriptive statistics, item analysis, content validity, exploratory factor analysis (EFA), and reliability analysis were conducted using IBM SPSS Statistics 26.0. Confirmatory factor analysis (CFA) was performed using IBM Amos 28.0. A value of *p* of less than 0.05 was considered statistically significant.

#### 2.4.1 Items analysis

Item analysis was conducted using the item-total correlation method, critical ratio method, and the calculation of Cronbach’s alpha coefficient and correlation coefficients for item selection. For the item-total correlation method: the relationship between each item score and the total score was calculated, with a higher r-value indicating better representativeness of the item. Any item with *r* < 0.4 or with a score difference from the total scale score that was not statistically significant (*p* > 0.05) was eliminated. Critical ratio method: the total scores were arranged in descending order, with the top 27% forming the high-score group and the bottom 27% forming the low-score group; differences between these groups were compared. The critical ratio (CR) is the *t*-value; if CR < 3 or the difference between the two groups was not statistically significant (*p* > 0.05), the item was deleted. Cronbach’s alpha coefficient and correlation coefficients: if removing an item resulted in a higher Cronbach’s alpha coefficient for the scale overall or a very small correlation coefficient, the item was deleted (29, 30).

#### 2.4.2 Validity analysis

Content validity indices include the item-level content validity index (I-CVI) and the scale-level content validity index/average (S-CVI/Ave). The I-CVI is calculated as the ratio of the number of experts rating an item as ‘3’ or ‘4’ to the total number of experts. The S-CVI/Ave is the average of all I-CVIs. Generally, an I-CVI of ≥0.78 and an S-CVI/Ave of ≥0.90 are considered indicative of good content validity of the scale (31, 32).

Structural validity was determined through exploratory factor analysis (EFA) and confirmatory factor analysis (CFA). The total sample was randomly divided into two parts (Sample 1 and Sample 2) using a simple random method, for use in EFA and CFA, respectively. Before conducting EFA, the suitability of the data for factor analysis was assessed using the Kaiser-Meyer-Olkin (KMO) measure and Bartlett’s test of sphericity. Generally, a KMO value >0.7 and Bartlett’s test *p*-value <0.01 are considered indicative of data suitability for factor analysis. If the data met the primary prerequisites for EFA, principal component analysis and varimax orthogonal rotation were further used to extract the number of common factors. A factor loading >0.4 was used as the criterion for attribution, with eigenvalues >1 and a cumulative percentage of variance explained by the factors >40% (33, 34).

In Confirmatory Factor Analysis (CFA), various indices such as the Normed Fit Index (NFI), Comparative Fit Index (CFI), Goodness of Fit Index (GFI), Incremental Fit Index (IFI), Tucker-Lewis Index (TLI), Root Mean Square Error of Approximation (RMSEA), and the Chi-square/degrees of freedom ratio (χ2/df) are utilized to assess the model’s fit and applicability. A model is deemed to have a good fit if NFI, CFI, GFI, IFI, and TLI are greater than 0.9, RMSEA is less than 0.08, and χ2/df is equal to or less than 3 (35, 36). For convergent validity, standardized factor loadings (λ), Construct Reliability (CR), and Average Variance Extracted (AVE) are measured. Typically, λ should be greater than 0.5 with *p* < 0.05, CR should be greater than 0.7, and AVE should be greater than 0.5. Discriminant validity is evaluated by comparing the square root of AVE to the absolute values of the correlations between factors, where the square root of AVE should be greater than these correlation coefficients (37, 38).

#### 2.4.3 Reliability analysis

Internal consistency reliability was assessed using Cronbach’s alpha coefficient, with a Cronbach’s alpha >0.7 considered indicative of good internal consistency (30). Split-half reliability was determined by dividing the sample into even and odd items, calculating the correlation coefficient r of the total scores for these two parts, and then correcting it using the Spearman-Brown formula R = 2r/(1 + r); a value >0.7 is generally acceptable (39). Test–retest reliability was assessed by retesting 30 participants after 2 weeks and calculating the intraclass correlation coefficient (ICC) between the scores of the two measurements; an ICC > 0.7 indicates good stability of the scale (30).

## 3 Ethical principle

This study was approved by the Ethics Committee of Linfen Central Hospital (approval number YP2023-57-1), and was conducted in accordance with the ethical standards set forth in the 1964 Declaration of Helsinki and subsequent amendments. All subjects signed a written informed consent form before participating in the study.

## 4 Results

### 4.1 General information

In this study, a total of 900 questionnaires were distributed, of which 14 were invalid, resulting in a response rate of 98.4%. A total of 886 women of childbearing age were recruited, with the majority being between 20 and 34 years old, accounting for 74.8%. Of these, 53.2% had urban household registration, 74.5% had a bachelor’s degree, and 59.9% were married. Additional sociodemographic information is presented in Table 1.

**Table 1:** General demography date (*n* = 886).

	*n*	%
Age		
15–19	33	3.7
20–24	212	23.9
25–29	244	27.5
30–34	207	23.4
35–39	129	14.6
40–44	42	4.7
45–49	19	2.1
Household registration		
City	471	53.2
Village	415	46.8
Education level		
Junior high school and below	27	3.0
High school/technical secondary school	12	1.4
Junior college	76	8.6
Undergraduate	660	74.5
Graduate student or above	111	12.5
Occupation		
Student	186	21.0
Be in employment	672	75.8
Unemployed	28	3.2
Marital status		
Spinsterhood	346	39.1
Married	531	59.9
Divorced	7	0.8
Other	2	0.2
Only child or not		
Yes	203	22.9
No	683	77.1
Medical insurance		
Yes	863	97.4
No	23	2.6
Average monthly earnings (CNY)		
<2000	58	6.5
2001–3,000	134	15.1
3,001–4,000	222	25.1
4,001–5,000	163	18.4
>5,000	309	34.9

### 4.2 Cross-cultural translation and pre-survey results of the scale

Based on expert opinions and after discussion by the research team, five revisions and improvements were made to the scale as follows: (a) Item 10 ‘Husband is overly attentive to me’ was modified to ‘Husband is overly attentive to me (e.g., excessive inquiry about feelings, over-regulation of actions and freedom, unnecessary care, etc.)’. (b) Item 18 ‘Dying due to pregnancy’ was changed to ‘Life being threatened due to pregnancy’. (c) Item 17 ‘Personal needs not being met’ was revised to ‘Personal needs (including physiological, psychological, and daily activities) not being met’. (d) Item 21 ‘Unable to establish a connection with the baby’ was altered to ‘Unable to establish a close relationship with the baby, such as through talking, feeling fetal movements, etc.’ (e) Items 29 ‘Unable to do household chores, such as cleaning and ironing’ and 30 ‘Unable to cook’ were merged to ‘Unable to perform household chores, such as laundry and cooking’. During the pilot study, participants reported that the items were easily understandable and clear. Therefore, no changes were made to the item content at this stage. This resulted in a Initial Chinese version of the FOPS with four factors and 29 items.

### 4.3 Item analysis

As indicated in Table 2, the Pearson correlation coefficient between Item 25 and the total scale score was 0.012 (*p* = 0.731), while the remaining 28 items had Pearson correlation coefficients ranging from 0.566 to 0.784 (*p* < 0.001). The Critical Ratio (CR) value for Item 25 was 1.543 (*p* = 0.123), and for the remaining 28 items, the CR values ranged from 14.384 to 30.656. The Cronbach’s alpha coefficient was 0.953. Upon deletion of any item, the Cronbach’s alpha for Item 25 was 0.957(>0.953), while the range for the remaining items was between 0.950 and 0.952(<0.953). Therefore, Item 25 was removed (29, 30), resulting in a final Chinese version of the FOPS with four factors and 28 items.

**Table 2:** Item analysis for Chinese version of the FOPS.

Item	Item-total correlation		Critical value method				Corrected item-total correlation	Cronbach’s alpha if item delete
	*r*	*p*	groups(Mean ± SD)		Critical ratio	*p*		
			Low score group (*n* = 242)	High score group (*n* = 240)				
1	0.630	<0.001	2.27 ± 1.519	4.33 ± 0.822	18.545	<0.001	0.598	0.951⬇
2	0.609	<0.001	2.55 ± 1.570	4.49 ± 0.797	17.108	<0.001	0.575	0.952⬇
3	0.623	<0.001	2.34 ± 1.441	4.33 ± 0.837	18.604	<0.001	0.591	0.952⬇
4	0.588	<0.001	2.71 ± 1.564	4.37 ± 0.877	14.384	<0.001	0.555	0.952⬇
5	0.657	<0.001	2.64 ± 1.603	4.66 ± 0.677	17.507	<0.001	0.628	0.951⬇
6	0.703	<0.001	1.43 ± 1.281	4.24 ± 0.807	29.331	<0.001	0.672	0.951⬇
7	0.694	<0.001	1.25 ± 1.166	4.02 ± 0.898	29.700	<0.001	0.664	0.951⬇
8	0.657	<0.001	1.51 ± 1.260	4.05 ± 1.048	24.092	<0.001	0.624	0.951⬇
9	0.683	<0.001	2.06 ± 1.542	4.60 ± 0.579	24.272	<0.001	0.651	0.951⬇
10	0.603	<0.001	1.43 ± 1.218	3.94 ± 1.223	22.369	<0.001	0.565	0.952⬇
11	0.707	<0.001	1.57 ± 1.344	4.45 ± 0.804	28.285	<0.001	0.677	0.951⬇
12	0.721	<0.001	1.47 ± 1.226	4.34 ± 0.807	30.656	<0.001	0.692	0.951⬇
13	0.724	<0.001	2.09 ± 1.511	4.50 ± 0.848	21.572	<0.001	0.698	0.951⬇
14	0.784	<0.001	2.04 ± 1.355	4.50 ± 0.670	25.923	<0.001	0.763	0.950⬇
15	0.769	<0.001	2.23 ± 1.424	4.62 ± 0.639	23.965	<0.001	0.748	0.950⬇
16	0.773	<0.001	1.82 ± 1.278	4.48 ± 0.753	27.579	<0.001	0.752	0.950⬇
17	0.742	<0.001	1.90 ± 1.273	4.49 ± 0.712	27.062	<0.001	0.718	0.950⬇
18	0.711	<0.001	2.18 ± 1.426	4.51 ± 0.696	22.953	<0.001	0.684	0.951⬇
19	0.742	<0.001	2.15 ± 1.296	4.41 ± 0.695	23.752	<0.001	0.720	0.950⬇
20	0.692	<0.001	1.97 ± 1.326	4.29 ± 0.906	22.170	<0.001	0.664	0.951⬇
21	0.703	<0.001	1.34 ± 1.032	4.03 ± 0.894	30.464	<0.001	0.674	0.951⬇
22	0.726	<0.001	1.36 ± 1.014	4.03 ± 0.912	30.202	<0.001	0.700	0.951⬇
23	0.762	<0.001	1.91 ± 1.347	4.39 ± 0.723	24.562	<0.001	0.740	0.950⬇
24	0.772	<0.001	1.93 ± 1.346	4.39 ± 0.726	24.758	<0.001	0.751	0.950⬇
25	**0.012**	**0.731**	2.89 ± 1.590	3.18 ± 1.770	**1.543**	**0.123**	**−0.044**	**0.957⬆**
26	0.620	<0.001	1.84 ± 1.435	4.38 ± 0.812	23.281	<0.001	0.583	0.952⬇
27	0.592	<0.001	1.95 ± 1.321	4.19 ± 0.834	21.588	<0.001	0.557	0.952⬇
28	0.576	<0.001	1.84 ± 1.398	4.08 ± 0.908	20.265	<0.001	0.539	0.952⬇
29	0.566	<0.001	1.54 ± 1.298	3.83 ± 1.115	20.833	<0.001	0.527	0.952⬇

Bold indicates that item 25 should be deleted; ⬆indicated increases: ⬇indicates decreases.

### 4.4 Validity analysis

#### 4.4.1 Content validity

The 12 experts involved in the cultural adaptation assessed the content validity of the Chinese version of the FOPS. The item-level content validity index (I-CVI) ranged from 0.833 to 1, while the scale-level content validity index/average (S-CVI/Ave) was 0.961.

#### 4.4.2 Exploratory factor analysis

The Kaiser-Meyer-Olkin (KMO) measure is 0.960, and Bartlett’s test of sphericity is significant (*χ*2 = 10460.254, df = 378, *p* < 0.001), indicating the data is suitable for factor analysis. Using Principal Component Analysis (PCA) and Varimax rotation, four common factors with eigenvalues greater than 1 were extracted. These factors collectively account for 72.578% of the total variance, as shown in Table 3.

**Table 3:** Exploratory factor analysis of the FOPS.

Factor loading	Factor 1	Factor 2	Factor 3	Factor 4	Common factor variance
15	0.861				0.806
23	0.861				0.805
16	0.853				0.797
14	0.842				0.806
24	0.828				0.775
13	0.816				0.713
19	0.811				0.739
22	0.796				0.697
18	0.789				0.683
17	0.753				0.654
21	0.742				0.632
20	0.730				0.602
7		0.877			0.846
6		0.871			0.835
11		0.867			0.832
12		0.820			0.775
10		0.771			0.645
9		0.766			0.664
8		0.703			0.633
5			0.807		0.778
2			0.800		0.725
1			0.787		0.716
3			0.699		0.626
4			0.695		0.554
26				0.845	0.818
29				0.839	0.779
27				0.782	0.704
28				0.751	0.682

F1, Maternal–infant health; F2, Spouse relationships; F3, Physical appearance; F4, Daily activities.

#### 4.4.3 Confirmatory factor analysis

**Figure 1:**
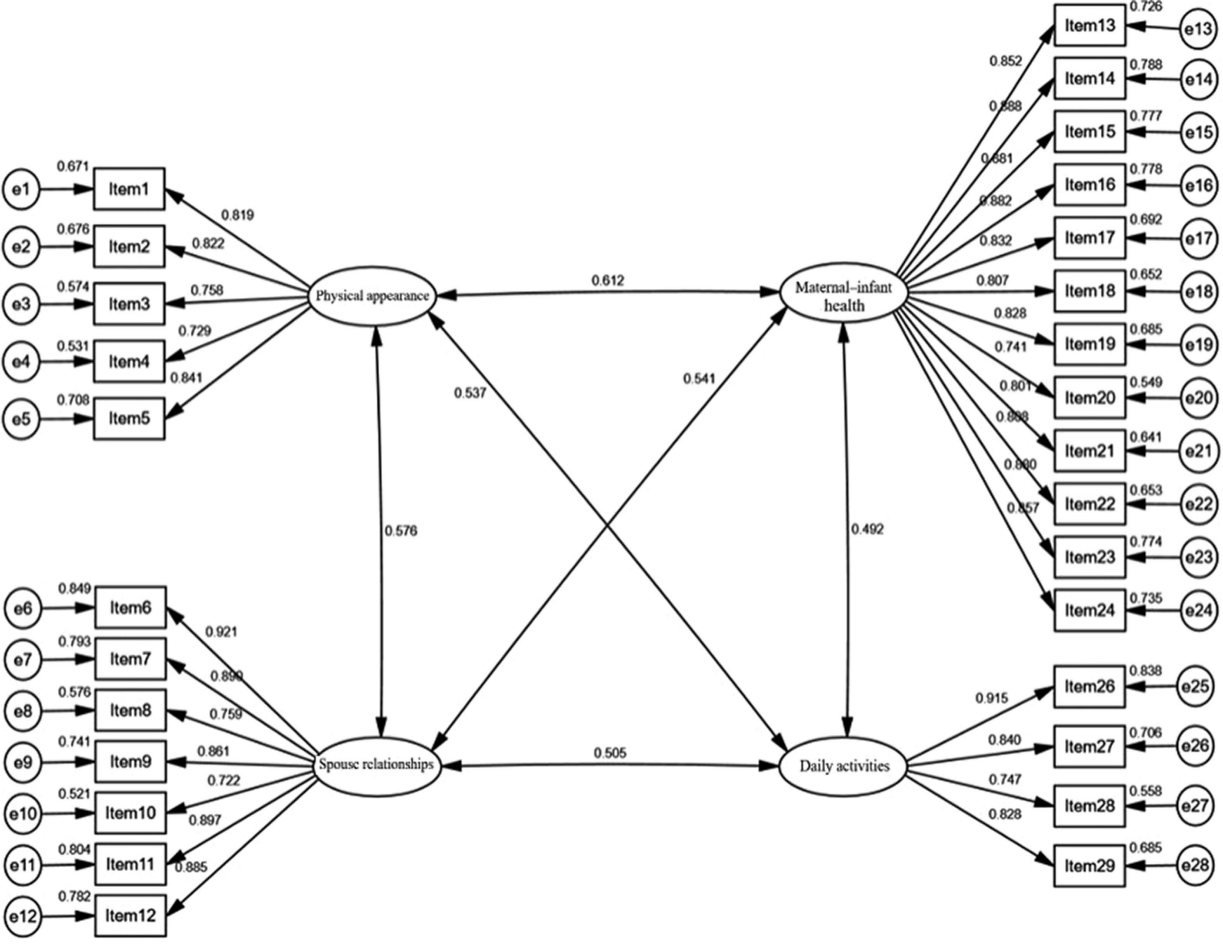
Standardized four-factor structural model of the Chinese version of FOPS.

A confirmatory factor analysis (CFA) model was established using the four factors from the exploratory factor analysis (EFA) as latent variables and the 28 items as manifest variables (Figure 1). The results of the model fit are presented in Table 4, indicating an overall good fit for the model. For convergent validity, the standardized factor loadings (λ) ranged from 0.722 to 0.921, all exceeding the 0.5 benchmarks, with a significance of *p* < 0.001, the Construct Reliability (CR) values for the four factors were 0.966, 0.948, 0.896, and 0.901, respectively, all >0.7. The Average Variance Extracted (AVE) values were 0.704, 0.724, 0.632, and 0.697, respectively, all >0.5. For discriminant validity, the square roots of AVE were all greater than the absolute values of the correlation coefficients between the factors (Table 5).

**Table 4:** Confirmatory factor analysis for the Chinese version of the FOPS.

Model fit indices	$\chi^{2}/df$	NFI	CFI	GFI	IFI	TLI	RMSEA
Four-factor model	1.444	0.956	0.986	0.927	0.986	0.985	0.032
Reference criteria	<3	>0.9	>0.9	>0.9	>0.9	>0.9	<0.08

**Table 5:** Aggregation validity and discriminant validity of the model.

Factor	Item	Convergent validity			Discriminant validity			
		*λ*	CR	AVE	F1	F2	F3	F4
F1	13	0.852***	0.966	0.704	0.839			
	14	0.888***						
	15	0.881***						
	16	0.882***						
	17	0.832***						
	18	0.807***						
	19	0.828***						
	20	0.741***						
	21	0.801***						
	22	0.808***						
	23	0.88***						
	24	0.857***						
F2	6	0.921***	0.948	0.724	0.541**	0.851		
	7	0.89***						
	8	0.759***						
	9	0.861***						
	10	0.722***						
	11	0.897***						
	12	0.885***						
F3	1	0.819***	0.895	0.632	0.612**	0.576**	0.795	
	2	0.822***						
	3	0.758***						
	4	0.729***						
	5	0.841***						
F4	25	0.915***	0.901	0.697	0.492**	0.505**	0.537**	0.835
	26	0.840***						
	27	0.747***						
	28	0.828***						

***p < 0.001, **p < 0.01. For discriminant validity, the bold numbers represent the correlation coefficients between factors, while the diagonal numbers are the square roots of the Average Variance Extracted (AVE) for each factor. It is observed that the diagonal numbers exceed the bold numbers, indicating good discriminant validity of the model. F1, Maternal–infant health; F2, Relationship with spouse; F3, Physical appearance; F4, Daily activities.

### 4.5 Reliability analysis

The Chinese version of the FOPS had an overall Cronbach’s alpha coefficient of 0.957, with the Cronbach’s alpha coefficients for each factor ranging from 0.887 to 0.965. The scale’s split-half reliability was 0.840, and its test–retest reliability was 0.932.

## 5 Discussions

### 5.1 Cross-cultural translation

This study strictly followed the Brislin translation-back translation model (28) for the cultural adaptation of the Fear of Pregnancy Scale into Chinese. During the translation process, careful consideration was given to the differences between English and Chinese, Turkish and Chinese, and English and Turkish. Multiple translation comparisons were conducted. Moreover, the translation team consisted of individuals with both medical and non-medical backgrounds. Through repeated comparative analyses, taking into account linguistic differences and the cultural context of China, the scale’s content, semantic, and conceptual equivalence, as well as its scientific accuracy, were enhanced.

Due to differences in language habits and cultural backgrounds across countries, cross-cultural adjustments were necessary to make the scale more compatible with Chinese culture and linguistic practices (40). Experts involved in the consultation came from various fields, including obstetrics, psychology, and nursing, and possessed extensive clinical, educational, and research experience, ensuring the content validity of the scale. Considering the experts’ opinions and after discussion by the research team, five revisions were made to the original scale in two aspects: (I) Modifications of expression: (a) Pregnant women generally prefer moderate attention from their husbands, but excessive concern can cause discomfort and stress (41). The original description was ambiguous, so Item 10 ‘Husband is overly attentive to me’ was changed to ‘Husband is overly attentive to me (e.g., excessive inquiry about feelings, over-regulation of actions and freedom, unnecessary care, etc.)’. (b) With advances in medical technology, deaths due to pregnancy are now very rare, almost nonexistent (42), so Item 18 ‘Dying due to pregnancy’ was revised to ‘Life being threatened due to pregnancy’. (c) Personal needs encompass various aspects, and the original statement was not specific enough, so Item 17 ‘Personal needs not being met’ was altered to ‘Personal needs (including physiological, psychological, and daily activities) not being met’. (d) The intimate relationship between a mother and her baby can include various aspects, so Item 21 ‘Unable to establish a connection with the baby’ was modified to ‘Unable to establish a close relationship with the baby, such as through talking, feeling fetal movements, etc.’ (II) Merging of items: (e) In China, ‘ironing’ is not a common household chore, and laundry and cooking are the usual household tasks, so Items 29 ‘Unable to do household chores, such as cleaning and ironing’ and 30 ‘Unable to cook’ were merged to describe ‘Unable to perform household chores, such as laundry and cooking’.”

### 5.2 Item analysis

Item analysis was conducted to assess and optimize the quality of the items in the scale. For Item 25, the Pearson correlation coefficient (r) was less than 0.4, and the Critical Ratio (CR) was less than 3. The Cronbach’s alpha coefficient increased to 0.957 after its removal, higher than the original 0.953, leading to its deletion (29, 30). Considering that the original scale included this item and categorized it under the ‘daily activities’ factor, the expert panel discussed this matter. The experts agreed that regret is an emotional experience, possibly parallel to, but not necessarily inclusive of fear, and thus should be removed. The remaining items had Pearson correlation coefficients (r) ranging from 0.566 to 0.784, and CR values from 14.384 to 30.656. The Cronbach’s alpha coefficients ranged from 0.950 to 0.952 after the removal of these items, indicating a high level of item discrimination (29, 30).

### 5.3 Validity analysis

Validity refers to the extent to which a measurement tool accurately reflects the target it is intended to measure. Content validity involves examining whether the content of a scale aligns with the research purpose and requirements. In this study, 12 experts from relevant fields were invited to evaluate the items of the scale using a 4-point Likert scale, ranging from 1 to 4, which represent ‘not relevant’, ‘somewhat relevant’, ‘quite relevant’, and ‘highly relevant’, respectively. After the expert evaluation, the item-level content validity index (I-CVI) and the scale-level content validity index/average (S-CVI/Ave) were obtained. It is considered that when the I-CVI and S-CVI/Ave values reach 0.78 and 0.90, respectively, the content validity of the scale is good. Items not meeting these criteria should be revised or deleted based on expert feedback. The results of this study showed that the I-CVI ranged from 0.833 to 1, and the S-CVI/Ave was 0.961, indicating that the scale items are representative and can accurately measure the concept of fear of pregnancy (31, 32).

Structural validity examines whether the relationship between the factors and measurement items aligns with expectations. In this study, the Kaiser-Meyer-Olkin (KMO) value was 0.960, and four factors were extracted, accounting for a cumulative variance contribution of 72.578%. The factor loadings for each item ranged from 0.685 to 0.877, with no cross-loadings observed (33, 34). The CFA results indicated good model fit, with NFI = 0.956, CFI = 0.986, GFI = 0.927, IFI = 0.986, TLI = 0.985, RMSEA = 0.032, and χ2/df = 1.444 (35, 36). Convergent validity, also known as aggregate validity, emphasizes that items belonging to the same factor indeed fall under that factor during measurement. The λ ranged from 0.722 to 0.921(*p* < 0.001), all exceeding the 0.5 benchmark, the CR values for the four factors were 0.966, 0.948, 0.896, and 0.901, all >0.7, and the AVEs were 0.704, 0.724, 0.632, and 0.697, all >0.5. Discriminant validity stresses that items not supposed to fall under the same factor are indeed separate during measurement. The square roots of AVE were greater than the absolute values of the correlation coefficients between the factors, indicating that internal consistency is greater than external consistency, suggesting distinctiveness among the latent variables and high discriminant validity (37, 38).

### 5.4 Reliability analysis

Reliability analysis is used to test the consistency, reliability, and stability of the results measured by a scale. Internal reliability is assessed using Cronbach’s alpha coefficient and split-half reliability, with a consensus that values >0.7 for both indicate good reliability. The overall Cronbach’s alpha coefficient for the Chinese version of the FOPS is 0.957, and the Cronbach’s alpha coefficients for the four factors range from 0.887 to 0.965. This indicates that the Chinese version of the FOPS and its factors have good internal consistency, higher than the overall Cronbach’s alpha coefficient (0.951) of the Turkish version. This improvement in reliability may be attributed to the deletion of Item 25, which had a lower correlation, thereby enhancing the consistency among items. The scale’s split-half reliability is 0.840, meeting the required standards and indicating good internal consistency and high reliability. Test–retest reliability measures the correlation of the results in the same group of subjects at two different times, with a minimum requirement of 0.7. The test–retest reliability of the scale is 0.932, demonstrating good external consistency and temporal stability of the scale (30, 39).

### 5.5 The Chinese version of FOPS is of great significance

China implemented its two-child policy on January 1, 2016, and its three-child policy on May 31, 2021. However, as of 2022, the number of newborns in China has declined for six consecutive years, with the newborn population decreasing by about 40% in the last 5 years. The total fertility rate in China has dropped to 1.09 in 2022, the lowest among countries with a population over a hundred million. The low fertility rate in China stems from a decline in the reproductive intentions of couples of childbearing age. In addition to increased economic pressure, higher child-rearing costs, and shifts in social attitudes, the fear of pregnancy among women (12, 43–45) may also be a significant factor contributing to the decline in fertility intentions. However, research on pregnancy fear among women of childbearing age in China is scarce, making an accurate, reliable, and culturally appropriate tool for measuring pregnancy fear crucial. Our study introduces the Fear of Pregnancy Scale to China, not only filling a gap in existing literature concerning the cross-cultural applicability of the scale but also providing a vital assessment tool for mental health professionals, obstetricians, and reproductive health researchers. Through this research, we aim to gain a deeper understanding of the characteristics of pregnancy fear in the Chinese cultural context, thereby better addressing the reproductive and mental health needs of women of childbearing age in China.

### 5.6 Limitations

This study has some limitations. First, to save time and resources, we employed convenience sampling in the communities of Linfen City, Shanxi Province, and Jinzhou City, Liaoning Province, and then used snowball sampling to reach a broader range of women of childbearing age through the social networks of the initial participants. Although this method allowed for wider coverage in these two regions, our sampling was confined to specific areas. Considering that regional economic conditions and lifestyles (8) can influence pregnancy fear, future research should involve multi-center, large-sample studies across the country. Secondly, the self-administered nature of the questionnaire may inevitably introduce bias. Future studies could employ semi-structured interviews for a more comprehensive understanding of the sources of pregnancy fear. Lastly, in this study, we did not explore the factors influencing pregnancy fear, which we plan to address in future research.

## 6 Conclusion

After translation and cross-cultural adaptation, the Fear of Pregnancy Scale has been introduced to China and demonstrates good reliability and validity. The Chinese version of the FOPS can assess the extent of pregnancy fear among Chinese women of childbearing age, understand the current status and influencing factors of pregnancy fear, and provide a theoretical basis for designing relevant policies and implementing targeted interventions in the prenatal healthcare system.

## Data availability statement

The raw data supporting the conclusions of this article will be made available by the authors, without undue reservation.

## Ethics statement

The studies involving humans were approved by the Ethics Committee of Linfen Central Hospital. The studies were conducted in accordance with the local legislation and institutional requirements. The participants provided their written informed consent to participate in this study.

## Author contributions

CW: Conceptualization, Investigation, Software, Validation, Writing – original draft. JZ: Conceptualization, Investigation, Methodology, Validation, Writing – original draft. LZ: Data curation, Investigation, Writing – original draft. YL: Data curation, Investigation, Writing – original draft. YY: Investigation, Writing – original draft. YW: Investigation, Writing – original draft. ZZ: Conceptualization, Methodology, Resources, Supervision, Writing – review & editing. SG: Conceptualization, Methodology, Resources, Supervision, Writing – review & editing.
